# Editorial

**Published:** 1859-09

**Authors:** 


### Editorial.

### THE FORMATION OF A NATIONAL SOCIETY.

The delegates appointed by the State and local Societies to meet at Niagara on the 3d ult., assembled at the appointed time. There were not much over half the delegates appointed present, but those who were there were good men and true, and were very unanimous as to the propriety and necessity of forming a National Association, that shall be thorough and effectual in its operations. So general was the opinion, that, without any hesitation, the initiatory steps were at once taken for the organization of a Society. Temporary officers were selected, and a committee appointed to draft a constitution. This was done, and the report presented to the meeting.—After some consideration of its provisions, it was referred back to the committee, with instructions to carefully revise and amend, if need be, and have it published through the Journals, within the next few months; and thus the profession will have ample opportunity to study its provisions. The final adoption will be at the next meeting, which will doubtless be done with great unanimity, as the draft presented is an excellent one, and all feel the importance of this organization. In addition to this, some work was laid out for the next meeting. Essayists were appointed, and their subjects designated, which are published in the proceedings. As they have one year in which to prepare this work, we expect it will be well done.

The meeting next year will consist of the same delegates, except where there are resignations. It would be well for the respective Societies to look a little to those delegates who were appointed, and did not attend, and either have some assurance that they will attend the next meeting, or appoint those who will. T.

### FANG FILLING OR NO FANG FILLING.

This subject will, from time to time, receive our attention in the pages of the Register, as space will permit and occasion may require. We will only now, and merely for his gratification, refer to one of Prof. M'Quillen's objections to our mode of practice. In the first number of the "Cosmos" he tells us that if the fangs are not filled, "the pulp cavity (though perfectly dry before the operation was performed) will soon be filled with serum through the foramen at the extremity of the fang." He calls in the aid of the microscope to prove his position, and announces—"as the *experimentum crucis*" —the remarkable fact that a thoroughly water-soaked tooth weighs more than a thoroughly dry one. We expect it does; and we like his theory, and would believe it if we could, and we know of no reason why we can not, except that it has no foundation in fact.—We will give but one case *now* to show our friend that his theory will not hold good.

A bicuspid tooth was filled after our method, April 16th, 1845. In the summer of 1853, the crown of the tooth was partially broken away, so that it became necessary to remove the filling, and refill. The canal was as dry as the powder of Cromwell's soldiers.

W.

### THE "COSMOS"

Has made its appearance. Not Humboldt's, good reader, but a real, genuine, North American Cosmos of our own. We refer to the "Dental Cosmos" of Messrs. Jones & White, and call it *our own,* because it is devoted to the interests of the great profession to which *we* belong.

The "Cosmos," as a monthly, succeeds the "News-Letter" as a quarterly, and makes a fine appearance, and is well filled with interesting matter. We expect in future numbers of the Register to have more room to notice the Cosmos, and will likely draw on it occasionally for our select, or review department. We like the Cosmos, and so do its publishers,—and so do its editors—most ardently we infer, as their enthusiasm on the subject *almost* runs away with their modesty. The publishers brag on the editors—one at a time, and the editors, with a single exception, brag on the publishers; and if we had room, we would brag on the whole of them, in real earnest; for they will all do to bet on, singly or combined. We wish the whole of them abundant success, and hope they will, according to promise, make the "Cosmos" more useful and successful than any journal that has yet preceded it in our country, and better than the Register—if they can. W.

### REPORT OF DISCUSSIONS.

In our report of the proceedings of the Convention at Niagara, we have endeavored to give that which would instruct—not that which was merely amusing. Some wit was displayed; but our readers can find just as genuine humor in a comic almanac or a cheap newspaper. Wit and humor enliven a public meeting, and often give point to the proceedings; but our space can be better occupied than in the publication of humorous sayings. The "laughter" and "applause" we have omitted, as usual. Our readers know as well what to applaud as the Convention did.

We believe that all the Journals had their "own reporters" at the Convention, and this will, doubtless, be found the best and most satisfactory way. Much time was uselessly spent with reference to the obtaining of an "official report," to be published in all the Journals. The brethren who figured in this movement seemed to forget that the Journals are private property, and might not wish to encumber their pages with such a report as the Convention might furnish. As long as the Convention remains a mere mass meeting, it has but little need of official reports. W.

### THE TRIP TO NIAGARA.

Nothing new can be said about railroads, and yet we feel like speaking a good word for that universal favorite, the LITTLE MIAMI. "We ought to praise the bridge that carries us safely over," (an original saying of ours,) and how much more ought we, then, to speak favorably of the road (bridges and all) that not only carries us safely, but snugly and quickly, over the smoothest possible track, in the most comfortable cars! We never feel like we were away from home, while in hearing of the whistle of this pioneer road; and our pride was more than once gratified in hearing our fellow-travelers express their surprise at the smoothness of the track.

Now, readers, if you want a pleasant and safe ride, just get tickets for Buffalo, via the Little Miami Railroad, and pay a visit to Niagara Falls; and you will not need our advice to induce you to go again. If not practicable sooner, we will hope to meet many of you on this route, on our way to the next Convention, at Saratoga.

Of Niagara itself, we have not one word to say. W.

### CRYSTAL TIN.

Some time ago a person, whose name I had never heard before, and can not remember since, called upon me, and proposed to give a formula for the preparation of crystal tin. The idea seemed a novel one, and perhaps from novelty, as much as from anything else, I obtained his formula. The experiments since lead me to think it a good thing, valuable in cases where tin is used for filling teeth. It is designed to take the place of tin foil. It is prepared as follows:

Take chemically pure hydrochloric acid, and dissolve tin (tin foil is best) in it till a saturated solution is obtained. This may be done speedily by heating the acid to a boiling point, or the same thing will be accomplished with the acid cold, in a few hours ; it is then muriate of tin. It is then poured into a clean vessel, and about an equal quantity of pure rain, or distilled water, added, then a clean, bright strip of sheet zinc is plunged into the solution, the tin is immediately deposited upon the zinc in a state of crystalization ; when a coating of desirable thickness is deposited upon the zinc, they may both be removed together from the solution, and the crystal tin slipped off the zinc into pure water ; the tin should be thoroughly cleansed, and again placed in the solution, to receive another coating of tin. It should be taken out and re-inserted, till all the tin is precipitated from the solution. The character of the crystalization will be modified somewhat by the extent of the dilution of the solution, in the first place. The tin should be thoroughly washed in pure water till all traces of the acid are gone, or a few drops of aquæ ammonia may be added to the water, to neutralize the acid. It has been suggested that it would be desirable to have a portion of the acid remaining in the tin, for filling teeth in which there is no sensitive dentine ; upon this, however, we have no experience. We have put in a few fillings of crystal tin—it works beautifully, and makes firmer fillings than foil. It must be kept in water, or what is probably better, alcohol. We think it preferable to tin foil—it works easier, unites more perfectly, and is absolutely pure tin, if well prepared. Try it. T.

---

EXCHANGES—COOL.

In almost all cases we get along smoothly with our exchanges. Occasionally we find them missing, either through the neglect of the Editors, Publishers, or Postmasters (not the Editors), most probably through the inefficiency or carelessness of the latter. We regard our exchanges too highly to slight or neglect any of them.

In two instances where we have sent the Register, and requested an exchange, we have received such treatment as only those who live in high latitudes give. A case we recently had may serve as an example. We sent the Register to a flourishing Journal, having a popular name, with a request to exchange ; said Journal is published at two dollars per year.

Had the publishers merely said they did not wish to exchange, we should have deemed it all right, and should not have uttered a word, nor entertained one thought about the matter; for certainly we do not wish to exchange with those who do not desire it.

Now, instead of notifying us that they did not wish to exchange, they sent us a prospectus, to be inserted in the Register, worth $10 per year, with the very modest suggestion, that if we will insert it, and give an *Editorial notice,* we shall have the *privilege* (*gracious?*) of exchanging with said Journal. How condescending! Wonder if the proposer is thawed out yet—guess not, for this happened in *dog days.* Old Sol may peel away on that fellow, without there being any probability of a *sun-stroke.* This proposition, though not of the same character, reminds us of a proposed trade, in which a certain personage agreed to sell all the world, when he did not own a single foot of it.

We don't want any better refrigerator than Mr. Q., even this hot weather. When we can't do without the Q., we will send it $2.00, and not give it $10 worth of advertising, to obtain the glorious privilege of an exchange. T.

